# Effects of crown-to-implant ratio on marginal bone level and bone density in non-splinted single implants: a cross-sectional study

**DOI:** 10.1186/s12903-023-03014-x

**Published:** 2023-05-16

**Authors:** Ziyuan Chen, Weiting Li, Peng Li

**Affiliations:** Second Clinical Division, Peking University School and Hospital of Stomatology & National Center for Stomatology & National Clinical Research Center for Oral Diseases & National Engineering Research Center of Oral Biomaterials and Digital Medical Devices, No.22, Zhongguancun South Avenue, Haidian District, Beijing, 100081 China

**Keywords:** Crown-to-implant ratio, Marginal bone level, Bone density, Non-splinted single implant

## Abstract

**Background:**

Few studies have evaluated the effects of the crown-to-implant (C/I) ratio on the marginal bone level (MBL) and bone density in non-splinted single implants. The aim of this study was to assess the effect of C/I ratio on MBL and density of peri-implant bone in non-splinted posterior implants.

**Methods:**

The C/I ratio, MBL, and grayscale values (GSVs) for bone density were measured from X-rays. Four areas of interest (two at the apical area and two at the middle of the peri-implant area) and two control areas were selected for evaluation. Follow-up radiographs were calibrated according to the control areas.

**Results:**

In all, 117 non-splinted posterior implants in 73 patients followed up for a mean duration of 36.23 ± 10.40 (range 24–72) months were considered. The mean anatomical C/I ratio was 1.78 ± 0.43 (range 0.93 to 3.06). The mean change in MBL was 0.28 ± 0.97 mm. There were no significant associations between the C/I ratio and MBL changes (*r* = -0.028, *p* = 0.766). Pearson correlation showed a significant correlation between changes in GSV and the C/I ratio in the middle peri-implant area (*r* = 0.301, *p* = 0.001) and apical area (*r* = 0.247, *p* = 0.009).

**Conclusions:**

A higher C/I ratio of single non-splinted posterior implants is associated with increased peri-implant bone density, but not correlated with changes in MBL.

## Background

Short implants are increasingly being used in cases with limited vertical bone dimensions to reduce surgical trauma and operative morbidity. Short implants have an intrabony length ≤ 8 mm [1]. However, some studies have found that an increased crown-to-implant (C/I) ratio negatively affects the biological interface between the bone and implant [2], resulting in marginal bone loss or enhanced mineralization of the peri-implant bone [3, 4].

Malchiodi et al. reported significantly greater crestal bone loss around implants with a higher C/I ratio [2]. However, Rossi et al. [5], Nunes et al. [6], and systematic reviews by Blanes [7] and Ravidà et al. [8] demonstrated that a high C/I ratio did not affect the peri-implant bone loss. However, various restoration designs have been included in the previous studies, without distinction between splinted and non-splinted crowns [9]. Therefore, conclusions could not be drawn.

Lee et al. [3] and Bulaqi et al. [4] found that a higher C/I ratio was associated with greater stress at the cortical peri-implant region. This mechanical stress can have positive or negative effects on the bone [10, 11]. Mechanical load is essential for bone remodeling. However, when the mechanical load exceeds the biological load-bearing capacity of the alveolar bone, tissue damage, including loss of osseointegration, occurs [10, 11]. Several studies have evaluated the effects of the C/I ratio on bone density and consequently failure rates. Only Sahrmann et al. reported a significantly higher peri-implant bone grey-scale values (GSVs) and level of mineralization around short implants than long implants [12].

In the present retrospective study, the effects of the C/I ratio on marginal bone level (MBL) and peri-implant bone density in non-splinted single implants were evaluated. The hypothesis was that a higher C/I ratio is associated with greater marginal bone loss and higher degree of mineralization of peri-implant bone.

## Methods

### Study population

This cross-sectional retrospective study was performed at the Second Clinical Division, Peking University School and Hospital of Stomatology between 2013 and 2020. The study protocol was approved by the Peking University School and Hospital of Stomatology Institutional Human Research Committee (protocol no. PKUSSIRB-202276071). And the study was conducted in accordance with the Declaration of Helsinki. Informed consent was obtained from patients via email or express email. The study included patients who had received press fit implants (Bicon Inc, Boston, MA, USA; width: 4.0–5.0 mm; length: 6.0–8.0 mm) in the posterior area that were supported by single non-splinted crown restorations and were followed for at least 24 months after loading. The implants were placed by an experienced periodontist (PL) and restored by two experienced prosthetic dentists with the same criteria as follows: the crown was cement-retained and the margin was located no deeper than 1 mm subgingivally; the excess cement was carefully removed. The medical records of patients were analyzed by an investigator (W.L), who also performed the radiographic measurements. The study results are reported in accordance with the Strengthening the Reporting of Observational Studies in Epidemiology (STROBE) guidelines.

### Follow-up examination

**Fig. 1:**
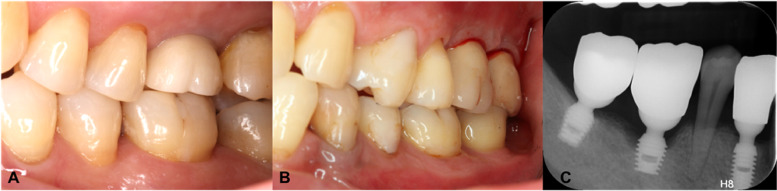
Peri-implant diseases and conditions. **A** Peri-implant health of the maxillary first molar. **B** Peri-implant mucositis with bleeding on probing of the maxillary molars. **C** Peri-implantitis with bone loss of the mandibular first molar

**Fig. 2:**
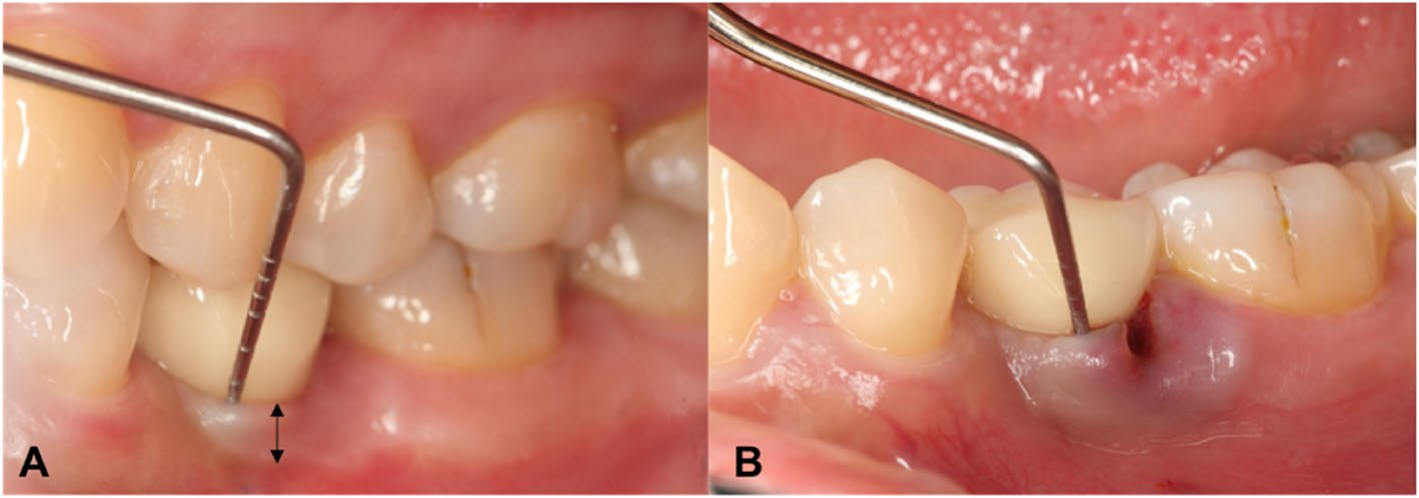
Clinical evaluation of probing depth and width of keratinized tissue. **A** Probing depth of 2 mm in healthy peri-implant mucosa. Black arrow: keratinized tissue at the mid buccal site. **B** Probing depth of 6 mm in inflammatory peri-implant mucosa

The restorations and implants were examined for signs of technical and biological complications. The biological complications included peri-implant mucositis and peri-implantitis (Fig. 1), diagnosed according to the 2017 World Workshop consensus report [13]. The technical complications included crown fracture or chipping, implant loosening or fracture, abutment loosening or fracture, and loss of crown retention. The following clinical parameters were recorded, using a UNC-15 periodontal probe (Hu-Friedy Mfg. Co., Chicago, IL, USA):

- Peri-implant probing depth (PDi) at six sites (Fig. 2).
- Peri-implant bleeding index (BIi): rated from 0 to 5 according to the Mazza Bleeding Index [14] at six sites.
- Peri-implant plaque index (PLIi): rated from 0 to 3 according to Mombelli et al. [15] at six sites.
- Width of keratinized tissue (WKT): at the mid-buccal point (Fig. 2).

### Radiographic analysis

Radiographs were obtained using a long-cone technique (INTR Soredex, Tuusula, Finland) at a voltage of 70 kV, explosion time of 0.16 s, and tube length of 22 cm. The digital images were analyzed for measurements.

**Fig. 3:**
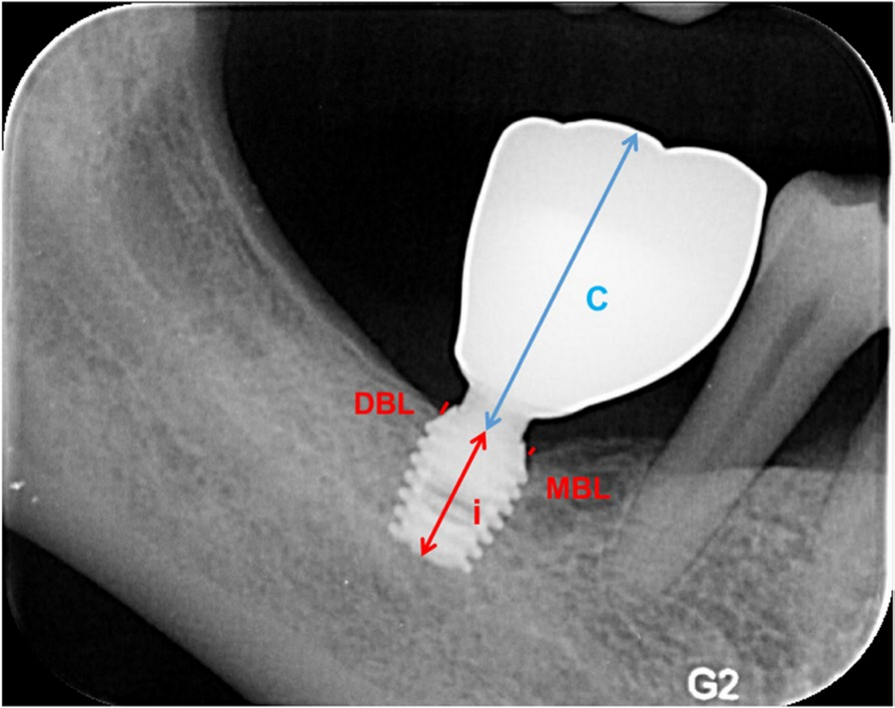
Implant length and marginal bone level measurements. C = crown length; i = implant length; DBL = distal bone level; MBL = mesial bone level

To calculate the anatomical C/I ratio, the implant length was measured from the apex to the implant shoulder (Fig. 3). The crown length was measured from the implant shoulder to the most occlusal point. The implants were divided into C/I ≥ 2 and < 2 groups [6, 7, 16] for comparison of outcomes.

### Marginal bone level changes

Bone level (BL) was defined as the distance between the implant platform and the first contact between the implant and bone (Fig. 3), and it was calibrated according to the actual implant length. The BL was measured at the mesial (M) and distal (D) aspects of each implant at baseline (initial bone level, IBL) and follow-up (final bone level, FBL). The change in MBL was calculated as the difference between the mean FBL and mean IBL. The following formulas were used:

- BL calibration: $$\mathrm{actual\ BL}=\frac{\mathrm{actual\ implant\ length}}{\mathrm{implant\ length}}*\mathrm{BL}$$  
- $$\mathrm{IBL}=\frac{\mathrm{IBL}(\mathrm{M})+\mathrm{IBL}(\mathrm{D})}{2}$$ and $$\mathrm{FBL}=\frac{\mathrm{FBL}(\mathrm{M})+\mathrm{FBL}(\mathrm{D})}{2}$$
- $$\mathrm{MBL\ change}=\mathrm{FBL}-\mathrm{IBL}$$  

### Bone density

**Fig. 4:**
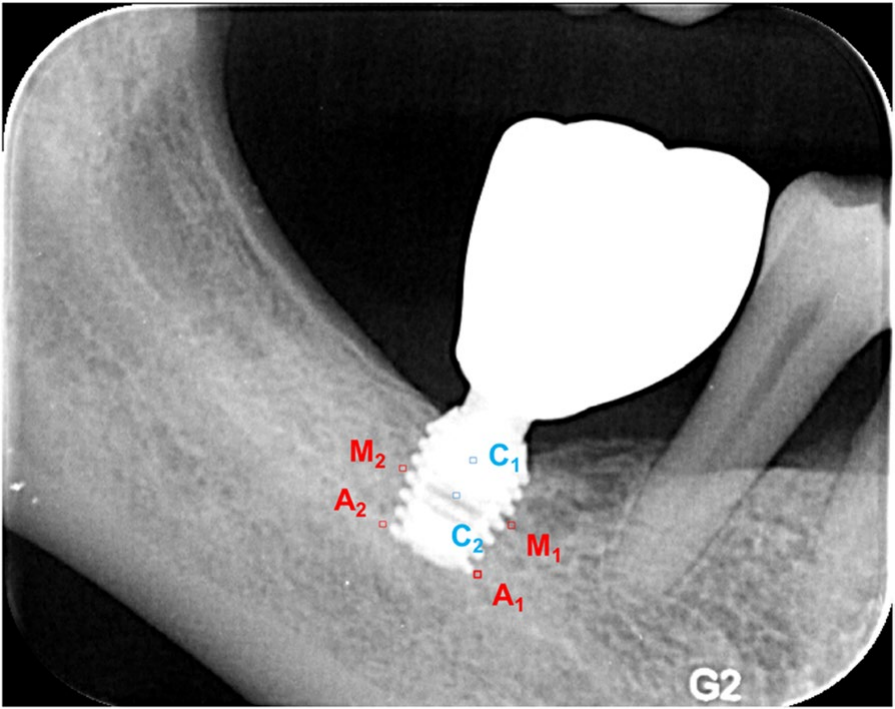
Bone density measurement. A1-2 = apical area; M1-2 = middle area; C1 = control area at the center of the abutment interface; C2 = control area next to the tip of the abutment interface

Six standardized assessment areas of interest (AOIs; 6 × 6 pixels) were set in each radiograph (Fig. 4). Two test AOIs M1 and M2 were placed at the peri-implant bone next to the mesial and distal implant surfaces at half-length of the implant, respectively [12]. Two additional test AOIs A1 and A2 were placed at the peri-implant bone next to the mesial and distal surfaces of the implant apex, respectively. Furthermore, two control AOIs C1 and C2 were placed for calibration. These AOIs were presumed to have a stable bone density during follow-up [12]. C1 was placed at the center of the abutment interface, and C2 was placed next to the tip of the abutment interface (Fig. 4).

The GSVs of the AOIs were analyzed using Digora for Windows (version 2.7; Digora, Hochfelden, France). The baseline and follow-up GSVs for the implants were presented as mean (M1 and M2), mean (A1 and A2) and mean (C1 and C2). A calibration factor (CF) was obtained by dividing the follow-up mean (FC1 and FC2) by the initial mean (IC1 and IC2). The CF was used to correct for possible changes in the ground brightness of photographs [12]. Finally, the difference in the GSVs (Δ GSV) of the AOIs (M, A) was calculated by subtracting the baseline GSV from the calibrated GSV obtained at the follow-up. The changes of bone density were represented by GSV changes. The following formulas were used:

- calibration factor: $$\mathrm{CF}=\frac{\mathrm{mean}(\mathrm{FC}1,\mathrm{FC}2)}{\mathrm{mean\ }(\mathrm{IC}1,\mathrm{ IC}2)}$$  
- $$\mathrm{calibrated\ GSV\ }(\mathrm{FM})=\frac{\mathrm{mean}(\mathrm{FM}1,\mathrm{FM}2)}{\mathrm{CF}}$$  
- $$\mathrm{calibrated\ GSV\ }(\mathrm{FA})=\frac{\mathrm{mean}(\mathrm{FA}1,\mathrm{FA}2)}{\mathrm{CF}}$$  
- $$\mathrm{changes\ in\ GSV\ }\left(\mathrm{M}\right)=\mathrm{calibrated\ GSV}\left(\mathrm{FM}\right)-\mathrm{mean}(\mathrm{IM}1,\mathrm{IM}2)$$  
- $$\mathrm{changes\ in\ GSV }\left(\mathrm{A}\right)=\mathrm{ calibrated\ GSV}\left(\mathrm{FA}\right)-\mathrm{mean}(\mathrm{IA}1,\mathrm{IA}2)$$  

### Statistical analysis

Statistical analysis was performed at implant level and patient level, using SPSS (version 24.0; IBM Corp., Armonk, NY, USA). *P* -values < 0.05 were considered indicative of statistical significance. Normally distributed quantitative data were compared between groups using independent-samples *t*-test (homogeneity) and corrected independent-samples *t-*test (no homogeneity). For non-normally distributed data, the Mann–Whitney U test was used. Qualitative variables were analyzed using the chi-square test. Pearson correlation was used to investigate the associations between the C/I ratio and changes in MBL and bone density.

## Results

This study included 73 patients (34 males and 39 females; mean age: 52.89 ±12.00 years, age range: 21-82 years) with 117 implants. The mean follow-up duration was 36.23 ± 10.40 (range: 24-72) months. In total, 11 (15.1%) patients were smokers and 3 (4.1%) had diabetes. All patients were treated for periodontitis and had remained on regular follow-up. The implants were placed after the periodontal treatment. No technical complications were recorded during the follow-up. However, three implants were diagnosed with peri-implantitis.

The mean anatomical C/I ratio was 1.78 ± 0.43 (range: 0.93–3.06). Overall, 31 (26.50%) and 86 (73.50%) implants had a C/I ratio ≥ 2 and < 2, respectively. Table 1 presents the baseline patient and implant characteristics. No significant differences were found between the two groups, except for the anatomical C/I ratio and implant location.

**Table 1:** Patient and implant characteristics

Variables		Anatomical C/I ratio ≥ 2	Anatomical C/I ratio < 2	*P*-value
**Baseline sample characteristics**				
**n**		18	55	
**Male/female**		10/8	24/31	0.379
**Age(y, mean ± SD)**		54.39 ± 8.65	52.40 ± 12.94	0.550
**Diabetes (n)**		0	3	0.570
**Smoking (n)**		3	8	> 0.99
**Baseline implant characteristics**				
**n**		31	86	
**GBR (n)**		1	2	> 0.99
**Follow-up (m, mean ± SD)**		35.03 ± 12.49	36.66 ± 9.51	0.457
**Anatomical** **C/I ratio (mean ± SD)**		2.33 ± 0.27	1.58 ± 0.29	< 0.001^*^
**Localization**	PM maxilla (n)	3	22	0.064
	PM mandible (n)	0	3	0.564
	M maxilla (n)	19	27	0.003^*^
	M mandible (n)	9	34	0.298
**Crown**	full monolithic zirconia (n)	29	84	0.612
	Porcelain (n)	2	2	
**Antagonist type**	Natural teeth (n)	25	78	0.248
	Implant (n)	4	5	0.381
	Removable (n)	1	1	0.461
	others (n)	1	2	> 0.99
**Outcome characteristics**				
**Peri-implant probing depth (mm, mean ± SD)**		3.73 ± 1.18	2.90 ± 0.71	0.001^*^
**Peri-implant bleeding index**		1.29 ± 0.93	0.90 ± 0.93	0.036^*^
**Peri-implant plaque index**		0.73 ± 0.63	0.67 ± 0.53	0.825
**Width of keratinized tissue**		3.42 ± 1.89	3.24 ± 1.37	0.639
**Marginal bone level** **Changes (mm, mean ± SD)**		0.24 ± 0.99	0.30 ± 0.97	0.848
${\varvec{\Delta}}$ **GSV M (grey, mean ± SD)**		5.10 ± 13.51	1.43 ± 12.84	0.295
${\varvec{\Delta}}$ **GSV A (grey, mean ± SD)**		-1.70 ± 15.21	-4.93 ± 16.89	0.494
**Peri-implantitis (n, %)**		1 (3.2%)	1 (1.2%)	0.461
**Peri-implant mucositis (n, %)**		23 (74.2%)	53 (61.6%)	0.209

*Abbreviations*: *C/I ratio* Crown-to-implant ratio, *GBR* Guided bone regeneration, *M* Molar, *PM* Premolar, $\Delta$ *GSV M* Changes of grey-scale value at middle peri-implant area, $\boldsymbol{\Delta }$ *GSV A* Changes of grey-scale value at apical peri-implant area

^*^P-value < 0.05

At the follow-up, the mean PDi (3.73 ± 1.18 mm) and BIi (1.29 ± 0.93) of the C/I ratio ≥ 2 group were significantly higher than those of the C/I ratio < 2 group (PDi: 2.90 ± 0.71 mm, *p* = 0.001; BIi: 0.90 ± 0.93, *p* = 0.036). However, there were no significant differences between the groups in terms of the PLIi and width of keratinized tissue (Table 1). Moreover, although the incidence of peri-implant mucositis of C/I ≥ 2 group (74.2%, 23/31) was higher than C/I < 2 group (61.6%, 53/86), the difference was not statistically significant (*p* = 0.209; Table 1).

The mean changes in MBL were 0.24 ± 0.99 and 0.30 ± 0.97 mm in the C/I ≥ 2 and < 2 groups, respectively, without significant difference between groups (*p* = 0.848). Pearson correlation analysis showed a nonsignificant correlation between the anatomical C/I ratio and changes in MBL (*r* = -0.028, *p* = 0.766; Table 2). Moreover, although the incidence of peri-implantitis of the C/I ≥ 2 group (3.2%, 1/31) was slightly higher than the C/I < 2 group (1.2%, 1/86), the difference did not reach statistical significance (*p* = 0.461; Table 1).

**Table 2:** The Pearson correlation analysis between anatomical crown-to-implant ratio and marginal bone level and bone density changes

Variables	mean ± SD	Pearson correlation	*P*-value
Anatomical C/I ratio	1.78 ± 0.43		
Marginal bone level changes, mm	0.28 ± 0.97	-0.028	0.766
${\varvec{\Delta}}$ GSV M, grey	2.38 ± 13.06	0.301	0.001^*^
${\varvec{\Delta}}$ GSV A, grey	-4.07 ± 16.45	0.247	0.009^*^

*Abbreviations*: *C/I ratio* Crown-to-implant ratio, $\Delta$ *GSV M* Changes of grey-scale value at middle peri-implant area, $\boldsymbol{\Delta }$ *GSV A* Changes of grey-scale value at apical peri-implant area

^*^P-value < 0.05

**Fig. 5:**
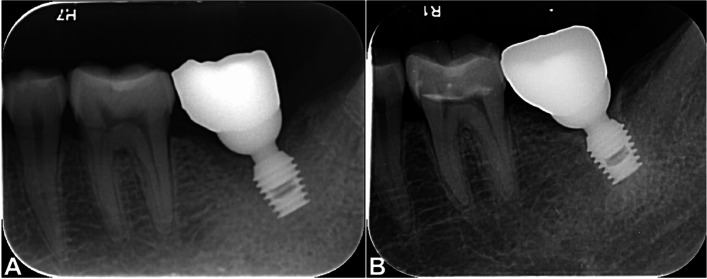
Peri-implant bone appears markedly denser around the implant after 3 years of loading. A. Initial radiograph. B. Radiograph after 3 years of loading

No significant differences were found between the groups in terms of the ${\varvec{\Delta}}$ GSV at the middle (M) or apical (A) peri-implant areas (Table 1). The Pearson correlation coefficient showed a significant correlation between ${\varvec{\Delta}}$ GSV M and the C/I ratio (*r* = 0.301, *p* = 0.001; Table 2), and between ${\varvec{\Delta}}$ GSV A and the C/I ratio (*r* = 0.247, *p* = 0.009; Table 2). Figure 5 shows a representative peri-implant bone with markedly increased density after 3 years of loading.

## Discussion

Although short implants are increasingly being used worldwide, the effects of an increased C/I ratio of single non-splinted posterior implants on the biological interface of bone and implant have not been evaluated. Therefore, the present study assessed the effects of the C/I ratio on changes in MBL and peri-implant bone density over a mean observation time of 36.23 ± 10.40 months. The results displayed that anatomical C/I ratio was significantly associated with bone density changes, but not correlated with changes in MBL.

Few clinical studies have evaluated the effect of the C/I ratio on bone density. The present research first found that a high C/I ratio was significantly associated with increased GSVs around the peri-implant bone. The outcomes are in line with Sahrmann et al. [12], who reported significantly increased GSVs around short implants but not long ones. However, they detected a non-significant association between the C/I ratio and GSVs in a regression analysis.

Panos Papaspyridakos et al. [17] found that short implants (≤ 6 mm) had higher variability and lower predictability of survival rates (86.7–100%) compared to longer implants (> 6 mm; 95–100%) after 1–5 years of loading. In clinical practice, we also noticed that some short implants lost bone osseointegration without prominent marginal bone loss, but demonstrated a pronounced brightening of the peri-implant bone in radiographs.

A bright appearance of bone in radiographs indicates a high degree of mineralization [18]. Finite element analysis has shown that an increased C/I ratio is associated with increased cortical bone stress [3, 4, 19]. In one study, when implant length was decreased from 15 to 8.5 mm, cortical bone stress increased [3]. In another study, decreasing the implant length had a moderate to large effect on increasing the stress and strain levels in the peri-implant cortical bone [19]. The increased mechanical stress promotes bone remodeling and mineralization, manifesting as increased bone-to-implant contact percentage and/or increased surrounding bone density [10, 11].

Under normal stress load, osseointegration is enhanced around the implant [20]. However, excessive stress leads to pathological resorption and fatigue microfracture. If these changes exceed the bone repair potential [10, 11], complete loss of osseointegration can occur in an already osseointegrated dental implant [11, 21]. In one animal study, after 18 months of excessive occlusal loading, six of the eight loaded implants lost osseointegration [22]. In Sahrmann et al. [12], a short-loaded implant was lost, without any clinical symtpoms of inflammation but with an obviously pronounced corticalization of peri-implant bone. When a new implant of regular length was placed at this site, it has been successfully loaded for 4 years [12]. Therefore, peri-implant bone mineralization indicates enhanced osseointegration or warning of bone over-stressing. Greater bone stress load can explain the higher variability of the survival rates of short implants.

In our analysis, a high anatomical C/I ratio did not affect the changes in MBL in non-splinted single implants, in line with previous studies [9, 21, 23–25]. Naenni et al. [21, 24] performed a randomized controlled clinical trial and reported similar changes in MBL between 6 mm long and 10 mm long single implants during a 5-year follow-up. Meta-regression analyses [9, 26] also did not detect a significant difference in implant loss rate and peri-implant bone level changes between different C/I ratio groups of single non-splinted implants.

In our study, although the groups did not show any significant differences in terms of changes in MBL and prevalence of peri-implantitis, peri-implant mucositis had a 10% higher prevalence in the C/I ≥ 2 group than in the < 2 group. In addition, the PDi and BIi were significantly higher in the C/I ≥ 2 group than in the < 2 group. Sahrmann et al. [21] also found a significantly higher number of implants with PD of ≥ 5 mm in 6-mm implant group than 10-mm group, at both baseline and follow-up. In our study, because the groups had similar PLIi and the cement residues could be removed as clear as possible by the slightly subgingivally located finish line (≤ 1.0 mm) [27], the different prevalence of peri-implant mucositis, PDi, and BIi between the groups can be explained by the differences in bone and soft tissue conditions at baseline. The C/I ratio ≥ 2 group generally had limited vertical bone dimensions, accompanied by excessive vertical soft tissue in patients with periodontitis [28]. And the peri-implant PDi was related to the vertical soft tissue thickness. The thicker the soft tissue thickness, the deeper PDi was detected [28]. Therefore, short implants in patient with periodontitis require frequent maintenance due to their higher risk for peri-implant mucositis.

Interestingly, both groups exhibited an increase in the mean MBL, consistent with previous studies of Bicon locking-taper implants [29, 30]. In Urdaneta et al. [29], 24.9% of implants showed crestal bone gain after an average of 70.7 months. Yoo et al. [30] documented crestal bone gain around immediately loaded locking-taper Bicon implants, with five implants gaining > 2 mm. Peri-implant bone gain has also been reported with other implant systems [31–34]. In Blanes et al. [31], 43.8% of ITI posterior dental implants exhibited bone gain after 5–10 years of follow-up. The bone gain may be attributable to the stimulation of the peri-implant bone remodeling by the loaded fixtures [35]. The load-bearing platform switching of Bicon implants converts the transcrestal portion of the implant-abutment complex into a load-transferring structure, which transmits compressive loads to the existing or potential crestal bone [36]. Therefore, the normal stress load can stimulate bone remodeling and gain.

There were a few limitations to the present study. First, it was retrospective and had a small sample size and short follow-up duration. Second, the bone density and MBL were measured from X-rays. Biopsies were not performed to assess exact histological bone quality and bone quantity. Third, although two calibration areas were used to minimize inaccuracies in the bone density measurements, two-dimensional radiographic assessments can be affected by image overlay. Nevertheless, the present study assessed the effects of the C/I ratio on bone density and MBL simultaneouly. To the best of our knowledge, this is the first study to identify an association between the C/I ratio and bone density of single non-splinted posterior implants.

## Conclusions

A higher C/I ratio of single non-splinted posterior implants is associated with higher bone density in peri-implant areas, but not correlated with changes in MBL.

## Data Availability

The datasets used and/or analysed during the current study are available from the corresponding author on reasonable request.

## Abbreviations

AOI: Areas of interest
BL: Bone level
BIi: Peri-implant bleeding index
C/I: Crown-to-implant ratio
FBL: Final bone level
GSV: Grey-scale value
IBL: Initial bone level
MBL: Marginal bone level
PDi: Peri-implant probing depth
PLIi: Peri-implant plaque index
WKT: Width of keratinized tissue
